# Supplementary material for: Cytoplasmic sequestration of p53 by lncRNA-CIRPILalleviates myocardial ischemia/reperfusion injury
Source: Commun Biol. 2022 Jul 18;5:716. doi: 10.1038/s42003-022-03651-y (PMC9293912; doi:10.1038/s42003-022-03651-y)

Supplementary information

Cytoplasmic sequestration of p53 by lncRNA-CIRPIL alleviates myocardial ischemia/reperfusion injury

Yuan Jiang[#, 1, 2, 3], Ying Yang[#, 1], Yang Zhang[#, 1], Jiqin Yang[1], Man-man Zhang[1], Shangxuan Li[1], Genlong Xue[1], Xingda Li[1], Xiaofang Zhang[1], Jiming Yang[1], Xiang Huang[1], Qihe Huang[1], Hongli Shan[4], Yanjie Lu[1], Baofeng Yang[1, 2, *], Zhenwei Pan[1, 2, *]

[1]Department of Pharmacology (State-Province Key Laboratories of Biomedicine-Pharmaceutics of China, Key Laboratory of Cardiovascular Research, Ministry of Education), College of Pharmacy, Harbin Medical University, Harbin, Heilongjiang 150086, P. R. China;

[2]Research Unit of Noninfectious Chronic Diseases in Frigid Zone, Chinese Academy of Medical Sciences, 2019 Research Unit 070, Harbin, Heilongjiang 150086, P. R. China;

[3]Department of Cardiology, Sun Yat-sen Memorial Hospital, Sun Yat-sen University, Guangzhou, Guangdong 510120, P. R. China;

[4]Shanghai Frontiers Science Research Center for Druggability of Cardiovascular noncoding RNA, Institute for Frontier Medical Technology, Shanghai University of Engineering Science, Shanghai, 201620, China.

[#]These authors contributed equally: Yuan Jiang, Ying Yang and Yang Zhang;

*Correspondence: Prof. Zhenwei Pan (panzw@ems.hrbmu.edu.cn) or Prof. Baofeng Yang (yangbf@ems.hrbmu.edu.cn). Department of Pharmacology, College of Pharmacy, Harbin Medical University, Baojian Road 157, Harbin, Heilongjiang 150081, P. R. China. Fax: 86 451 86675769 or 86667511 Tel: 86 451 86671354

Supplementary Tables

Supplementary Table 1. Bioinformatics analysis of lncCIRPIL's coding potential basing on the analysis of the Coding Potential Calculator 2(CPC2) database (http://cpc2.cbi.pku.edu.cn).

Fickett score	putative ORF length	ORF integrity	Isoelectric point
0.33323	86 aa	Completely	9.68719482422

Supplementary Table 2. Principal echocardiography parameters.

**Group**	**WT (n=10)**	**LncCIRPIN(TG) (n=9)**	**LncCIRPIL (KO) (n=9)**	**I/R+WT (n=11)**	**I/R+LncCIRPIL(TG) (n=11)**	**I/R+LncCIRPIL(KO) (n=9)**
EF, %	71.91±0.70	72.55±0.89	71.74±0.90	52.57±0.58 [a]	61.17±1.07 [b]	42.61±1.60 [b]
FS, %	40.09±0.60	40.64±0.71	39.59±0.73	26.41±0.37 [a]	32.08±0.75 [b]	20.53±0.92[b]
LVEDV, μl	44.88±1.47	45.80±2.87	44.33±1.32	56.57±2.20 [a]	51.30±1.27	56.87±2.47
LVESV, μl	12.59±0.48	12.67±1.05	12.56±0.61	26.82±1.06 [a]	19.87±0.59 [b]	32.55±1.39 [b]
LVIDd, mm	3.32±0.05	3.34±0.09	3.30±0.04	3.65±0.06 [a]	3.51±0.04	3.66±0.06
LVIDs, mm	1.99±0.03	1.98±0.07	1.99±0.04	2.69±0.04 [a]	2.38±0.03 [b]	2.91±0.05 [b]

[a]$p < 0.05$ versus WT group; [b]$p < 0.05$ versus I/R+WT group.

They were analyzed by using two-way ANOVA, followed by Bonferroni's post hoc analysis. The data are expressed as means ± SEM.

Supplementary Table 3. Sequences of shRNAs and siRNAs.

Name	Target sequence
LncRNA-CIRPIL (shRNA-1)	5'- CGCCTCAAAGAATAAGGCAGA -3'
LncRNA-CIRPIL (shRNA-2)	5'- GCAGAACAGACTGGACAGGAA -3'
LncRNA-CIRPIL (shRNA-3)	5'- GTCACCAGATGTGACAGGAAA -3'
COP1(shRNA-1)	5'- GCAGTGTCTTATGCCAAGTTT -3'
COP1(shRNA-2)	5'- GCTGTATCAGTTGGAGTAGTT -3'
COP1(shRNA-3)	5'- GCAAGCCAGTTAGATGAATTT- 3'
CHIP(shRNA-1)	5'- CCCTTCGCATTGCTAAGAAGA -3'
CHIP(shRNA-2)	5'- GAGAGTTATGATGAGGCCATT -3'
CHIP(shRNA-3)	5'- GAGAGTGAGCTGCATTCATAT -3'
MDM2(shRNA-1)	5'- CCAATCCAAATGATTGTGCTA -3'
MDM2(shRNA-2)	5'- GTGACGACTATTCCCAACCAT -3'
MDM2(shRNA-3)	5'- GTGTACCTCATGCAATGAAAT -3'
HUWE1(shRNA-1)	5'- CCGCACTGTGTTAAACCAGAT -3'
HUWE1(shRNA-2)	5'- CCACAAATATGCCATGATGTT -3'
HUWE1(shRNA-3)	5'- GCACTGCTCATCAAAGATGTT -3'
RCHY1(shRNA-1)	5'- ACTCCCATGCCATCCGAATAC -3'
RCHY1(shRNA-2)	5'- CTCCTACATAGAACGTGTTAT -3'
RCHY1(shRNA-3)	5'- CCGGCAGAATTGTCCAATATG -3'
p53(siRNA-1)	5'- GGACAGCCAAGTCTGTTAT -3'
p53(siRNA-2)	5'- GACCTATCCTTACCATCAT -3'
p53(siRNA-3)	5'- CCACTTGATGGAGAGTATT -3'

NC(siRNA)	5'- TTCTCCGAACGTGTCACGT -3'

Supplementary Table 4. The sequence of primers for Real-Time PCR

Primer name	Primer sequence
p53 (mouse)	Forward: 5'-TGGAGGAGTCACAGTCGGAT-3'<br>Reward: 5'-CAGTGAGGTGATGGCAGGAT-3'
Actin (mouse)	Forward: 5'-GACAGCAGTTGGTTGGAGCA-3'<br>Reward: 5'-TTGGGAGGGTGAGGGACTTC-3'
Bax (mouse)	Forward: 5'-TGGAAGAAGATGGGCTGAGG-3'<br>Reward: 5'-TTCCCACCCCTCCCAATAAT-3'
PUMA (mouse)	Forward: 5'-AGCAGCACTTAGAGTCGCC-3'<br>Reward: 5'-CCTGGGTAAGGGGAGGAGT-3'
CHIP (mouse)	Forward: 5'-CCATCACTCGGAACCCACTTG-3'<br>Reward: 5'-TGGCCTCATCATAACTCTCCA-3'
MDM2 (mouse)	Forward: 5'-TGTCTGTGTCTACCGAGGGTG-3'<br>Reward: 5'-TCCAACGGACTTTAACAACTTCA-3'
COP1 (mouse)	Forward: 5'-TAGCAGTAGGAAGCGACCTCT-3'<br>Reward: 5'-TGCCTCCTCAATCATATCAAAGC-3'
HUWE1 (mouse)	Forward: 5'-ACTGCATTCAGGCCATGATTG-3'<br>Reward: 5'-TGATAAGGTCAACCACTCTGACA-3'
RCHY1 (mouse)	Forward: 5'-GGCTGTGTCACGATACCAATG-3'<br>Reward: 5'-GTGCTACAGTCTTCACAAGTCTG-3'
LncRNA-CIRPIL (mouse)	Forward: 5'-ATATCCAGAGGAAAGGCGTCC-3'<br>Reward: 5'-TGACTCCTCGACCTGACTGA-3'
LncRNA-CIRPIL (human)	Forward: 5'-GGCGTCTATAATTAGGCCCT -3'<br>Reward: 5'-TGGCACGCAGTGAACGATAG-3'
p53 (human)	Forward: 5'-CAGTCTACCTCCCGCCATAA-3'<br>Reward: 5'-GCTTCTGACGCACACCTATT-3'
GAPDH (human)	Forward: 5'-CATGTTCGTCATGGGTGTGAA-3'<br>Reward: 5'-GGCATGGACTGTGGTCATGAG-3'

Supplementary Table 5. Information of Antibodies used in the study.

Antibody	Dilution	Company	Catalog Number
p53	WB 1:1000<br>IF 1:500<br>IP 1:200	Cell Signaling Technology	2524
p53	IP 1:200<br>WB 1:1000	Proteintech	10442-1-AP
Bax	WB 1:1000	Proteintech	50599-2-lg
Bcl2	WB1:1000	Abclonal	A19693
β-actin	WB 1:1000	Cell Signaling Technology	4970
Lamin-B	WB 1:1000	Abclonal	A1910
LC3I/II	WB 1:1000	Cell Signaling Technology	4108
DYKDDDDK Tag	WB 1:1000<br>IP 1:200	Cell Signaling Technology	2368
α-actin	IF 1:500	Sigma-Aldrich	A2522
IRDye 700CW goat anti-rabbit IgG(H+L)	WB 1:10000	Licor	926-68021
IRDye 800CW goat anti-mouse IgG(H+L)	WB 1:10000	Licor	926-32210
IRDye 800CW goat anti-rabbit IgG(H+L)	WB 1:10000	Licor	926-32211

Supplementary Table 6. The binding potential analysis of lncCIRPIL and p53 by using the RNA-Protein Interaction Prediction (RPISeq) database **(http://pridb.gdcb.iastate.edu/RPISeq/).**

Interaction probabilities<br>(Prediction using RF classifer)	0.7
Interaction probabilities<br>(Prediction using SVM classifer)	0.6

The predictions with probabilities > 0.5 were considered “positive

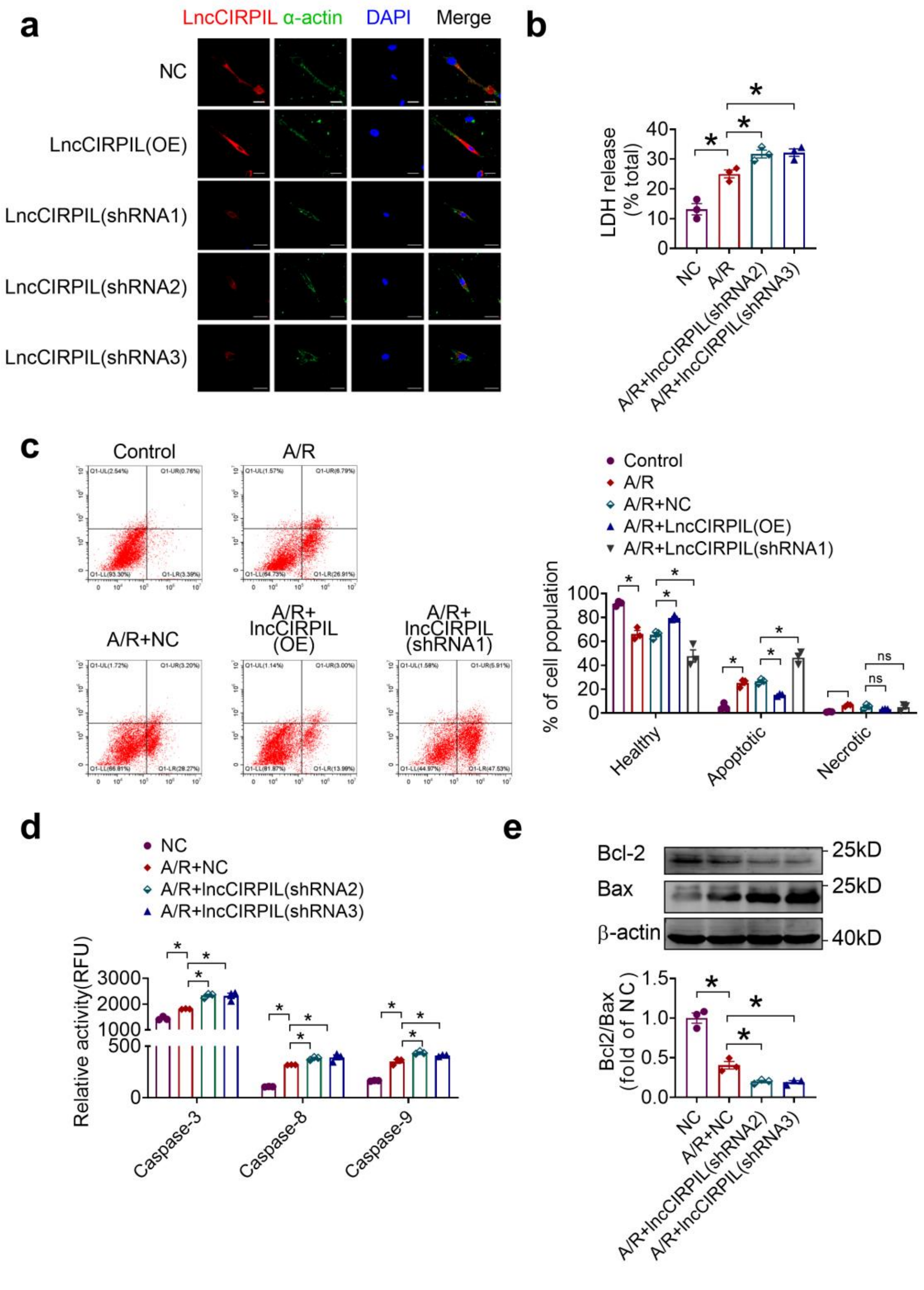

Supplementary Figure 1. The efficiency of overexpression and knockdown lncCIRPIL by lncCIRPIL overexpression and shRNA plasmid. **a.** Transfection efficiency of lncCIRPIL overexpression plasmid and shRNAs in NMCMs by Fluorescent in situ hybridization (FISH) & Immunofluorescence (IF) after A/R or I/R injury. n = 3. Scale bar = 20 μm. Red, lncCIRPIL, green, α-actin; blue, 4',6-

diamidino-2-phenylindole (DAPI). **b.** Effects of shRNA2 and shRNA3 of lncCIRPIL on LDH release from NMCMs subjected to A/R insult. n = 3. *P < 0.05 by one-way ANOVA followed by Tukey post hoc analysis. **c.** Cell death stained by annexin V and propidium iodide (PI) was determined using flow cytometry. n = 3. *P < 0.05 by one-way ANOVA followed by Tukey post hoc analysis. **d.** Effects of shRNA2 and shRNA3 of lncCIRPIL on Bcl2/Bax ratio in NMCMs subjected to A/R insult. n = 3. *P < 0.05 by one-way ANOVA followed by Tukey post hoc analysis. **e.** Effects of shRNA2 and shRNA3 of lncCIRPIL on caspase-3, caspase-8, caspase-9 activity in NMCMs subjected to A/R insult. n = 3. *P < 0.05 by one-way ANOVA followed by Tukey post hoc analysis.

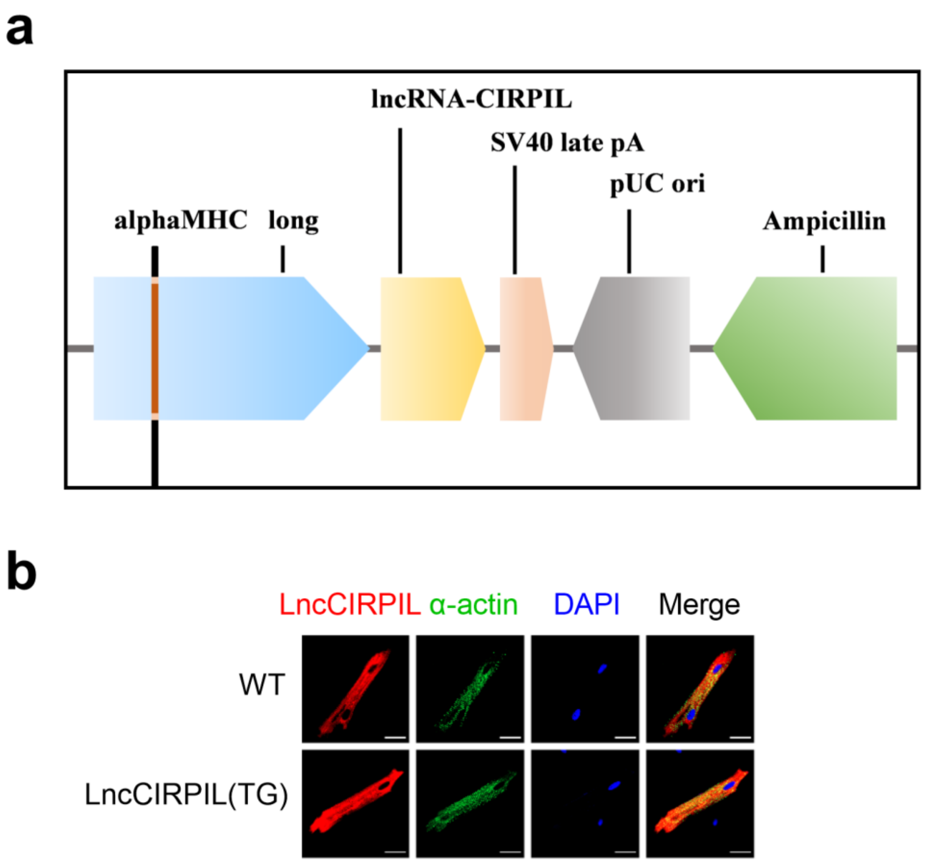

Supplementary Figure 2. Generation and verification of cardiac-specific lncRNA-CIRPIL transgene mice. **a.** Strategy for the generation of cardiac-specific lncRNA-CIRPIL transgene mice. The sequence of lncRNACIRPIL was cloned into the murine α-MHC promoter expression vector and the obtained DNA fragment containing lncRNA-CIRPIL driven by α-MHC promoter was microinjected into fertilized eggs. **b.** Expression of lncCIRPIL in adult cardiomyocytes of lncCIRPIL transgenic mice by FISH & IF assay. n = 3. Scale bar = 20 μm. Red, lncCIRPIL, green, α-actin; blue, DAPI.

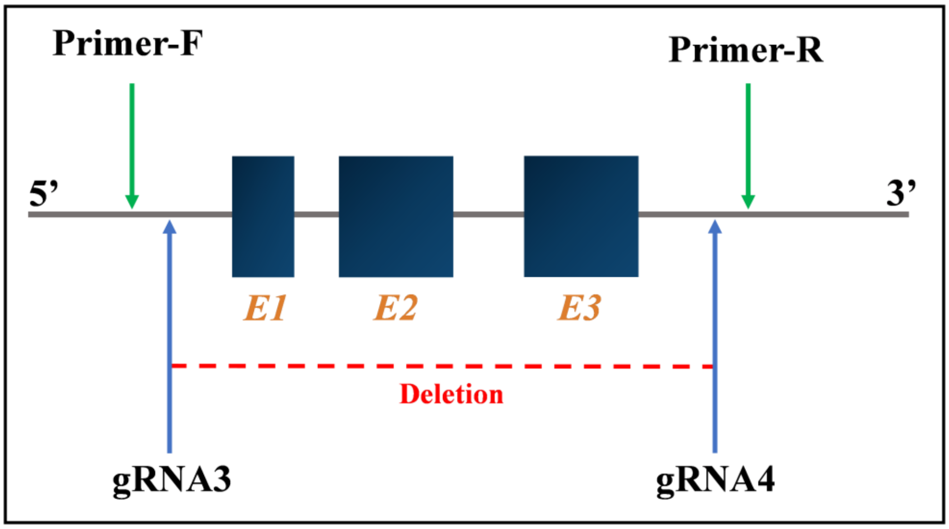

Supplementary Figure 3. Strategy for the deletion of lncCIRPIL by CRISPR/Cas9 technique. The chr5 111490827-111492382 gene is located on mouse chromosome 5. The three exons were completely ablated.

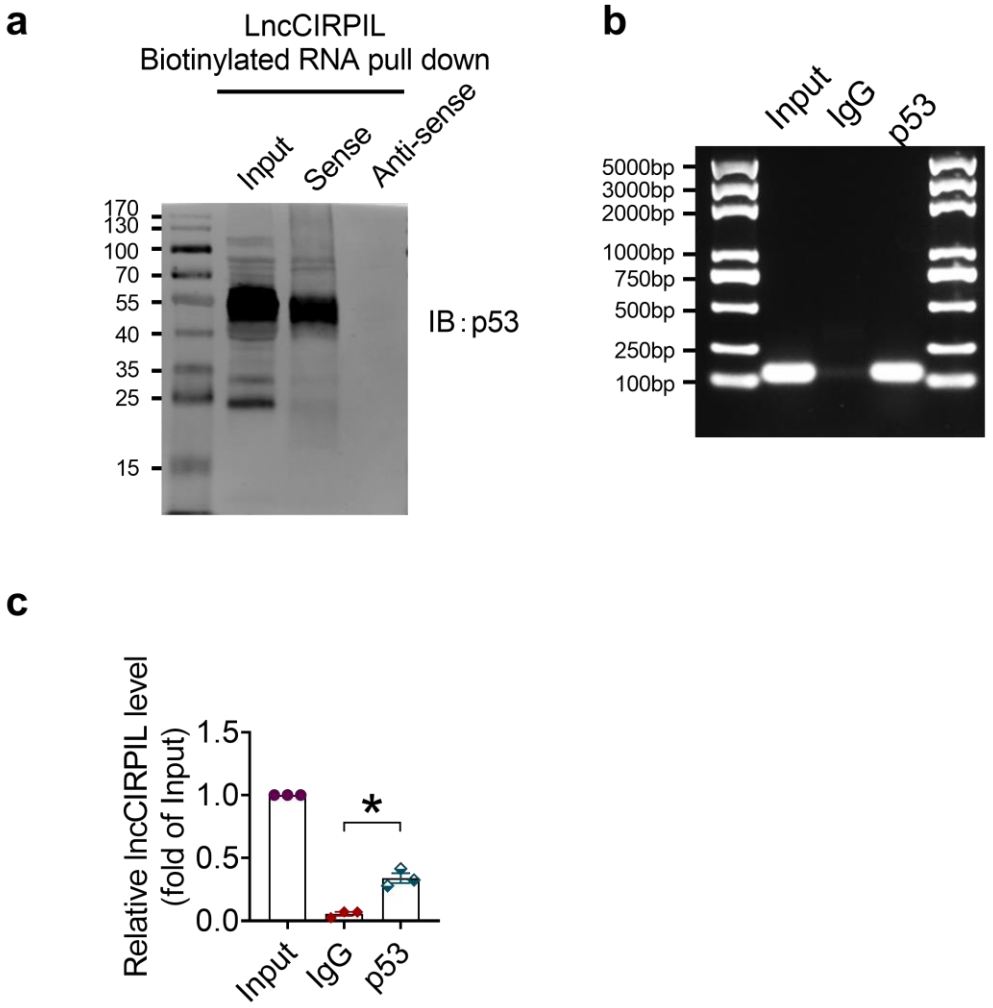

Supplementary Figure 4. The analysis for the binding potential of lncCIRPIL and p53. **a.** Western blot of p53 pulled down by sense sequence of lncCIRPIL in heart tissue of mice. n = 3. **b.** Image of PCR products of RNA immunoprecipitation (RIP) assay. **c.** Statistical PCR data of lncCIRPIL. n = 3. *$P < 0.05$ vs IgG by two-tailed Student's *t* test.

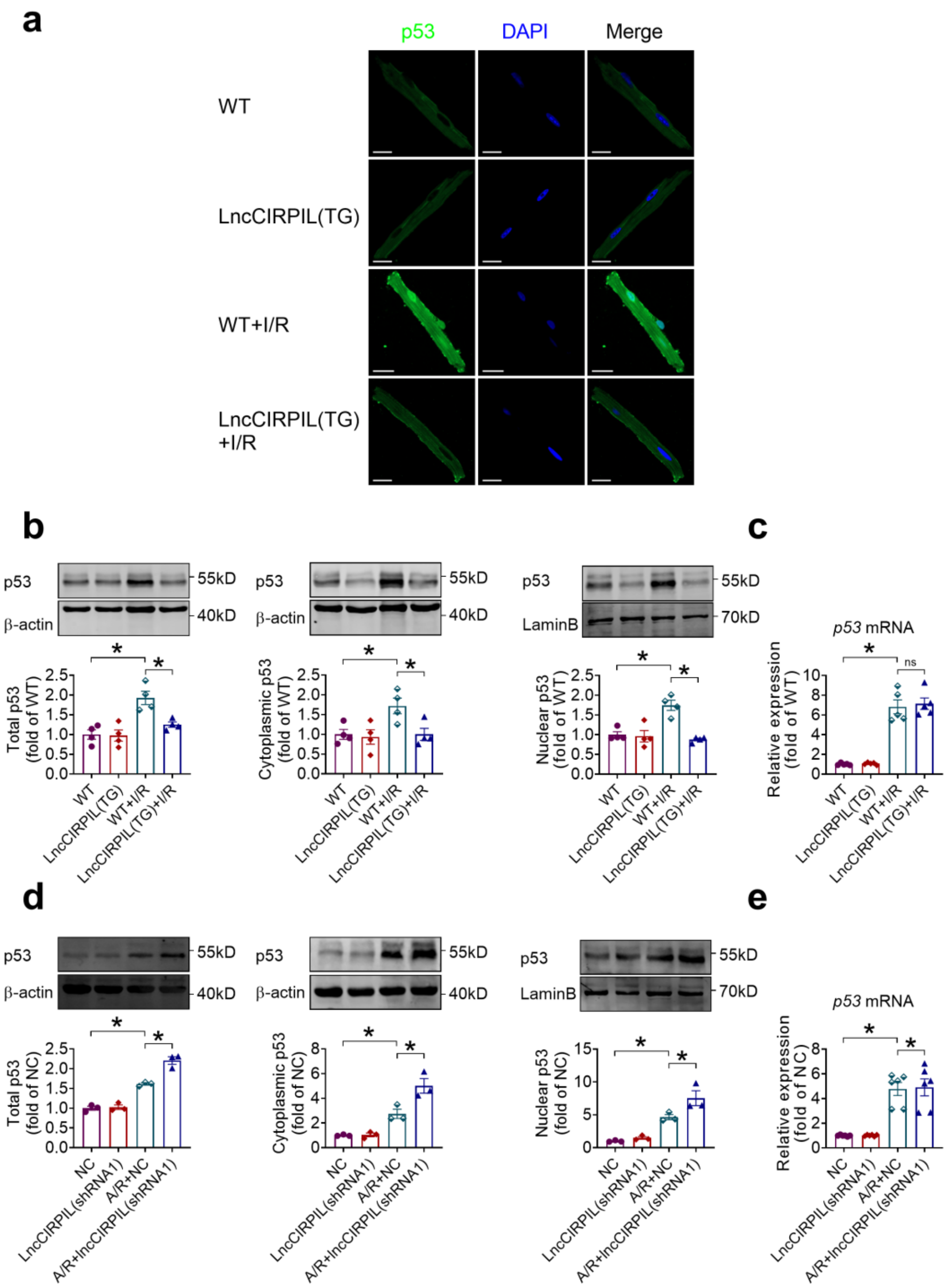

Supplementary Figure 5. Effects of lncCIRPIL on p53 expression and distribution in mice detected. **a.** Cytoplasmic and nuclear distribution of p53 protein in isolated adult cardiomyocyte from WT and lncCIRPIL TG mice subjected to I/R injury by immunofluorescence staining. n = 5. Scale bar = 20 μm. **b.** The total, cytoplasmic and nuclear levels of p53 in hearts of WT and lncCIRPIL-TG mice subjected to I/R injury.

n = 4. *$P < 0.05$ by one-way ANOVA followed by Tukey post hoc analysis. **c.** The *p53* mRNA levels in the hearts of WT and lncCIRPIL TG mice subjected to I/R injury. n = 5. *$P < 0.05$ by by one-way ANOVA followed by Tukey post hoc analysis. **d.** The effects of lncCIRPIL knockdown on total, cytoplasmic and nuclear levels of p53 protein in NMCMs subjected to A/R insult. n = 3. *$P < 0.05$ by one-way ANOVA followed by Tukey post hoc analysis. **e.** The effect of lncCIRPIL knockdown on *p53* mRNA level of NMCMs subjected to A/R insult. n = 9 samples from 3 independent experiments for each group. *$P < 0.05$ by one-way ANOVA followed by Tukey post hoc analysis.

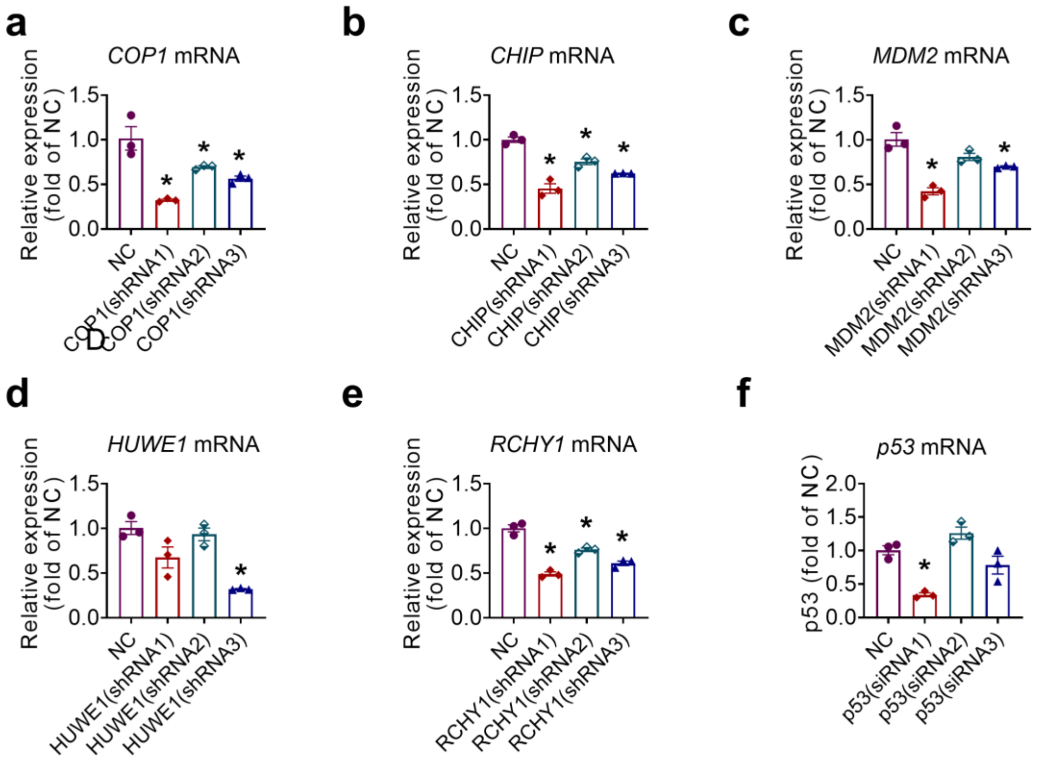

Supplementary Figure 6. Knocking down efficiency of shRNAs for E3 ubiquitin ligases**. a-f.** Knocking down efficiency of shRNAs for COP1, CHIP, MDM2, HUWE1, RCHY1 and siRNAs for p53 in NMCMs. n = 3 for each group. **P* < 0.05 vs NC by two-tailed Student's *t* test.

a

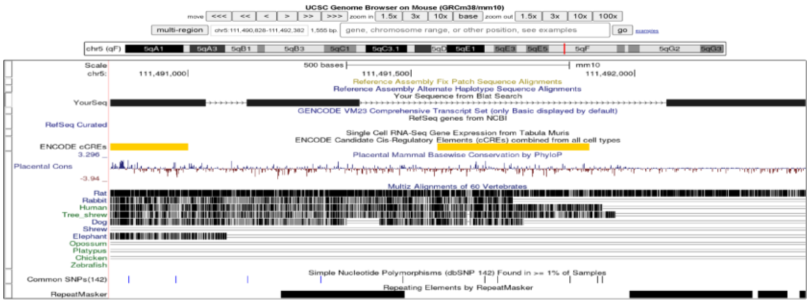

b

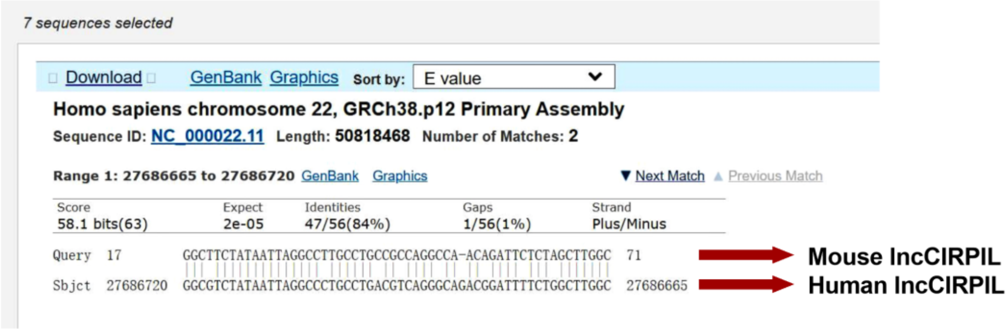

Supplementary Figure 7. The analysis of conserved regions of lncCIRPIL. **a.** The genomic locus of lncCIRPIL indicating the genomic conserved regions across species (http://genome.ucsc.edu). **b.** Pair-wise alignment by BLAST indicating a high degree conservation region of lncCIRPIL across human and mouse.

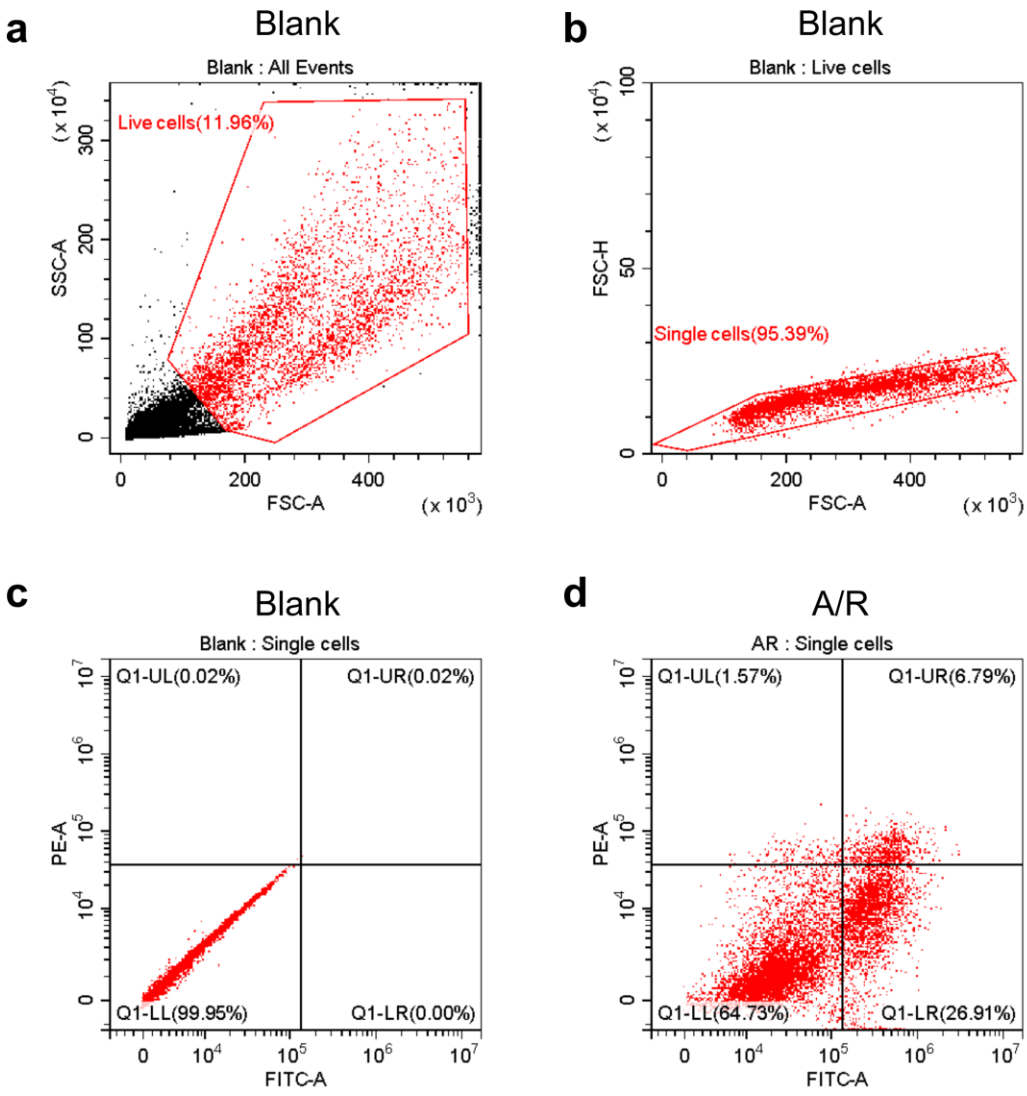

Supplementary Figure 8. Flow cytometry gating strategy for cell death detection by Annexin V-FITC and PI staining. **a-d.** Three cell subpopulations identified from the singlet population of Annexin V-FITC & PI stained A/R cells with unstained control cells as reference.

Unprocessed images of western blot assay

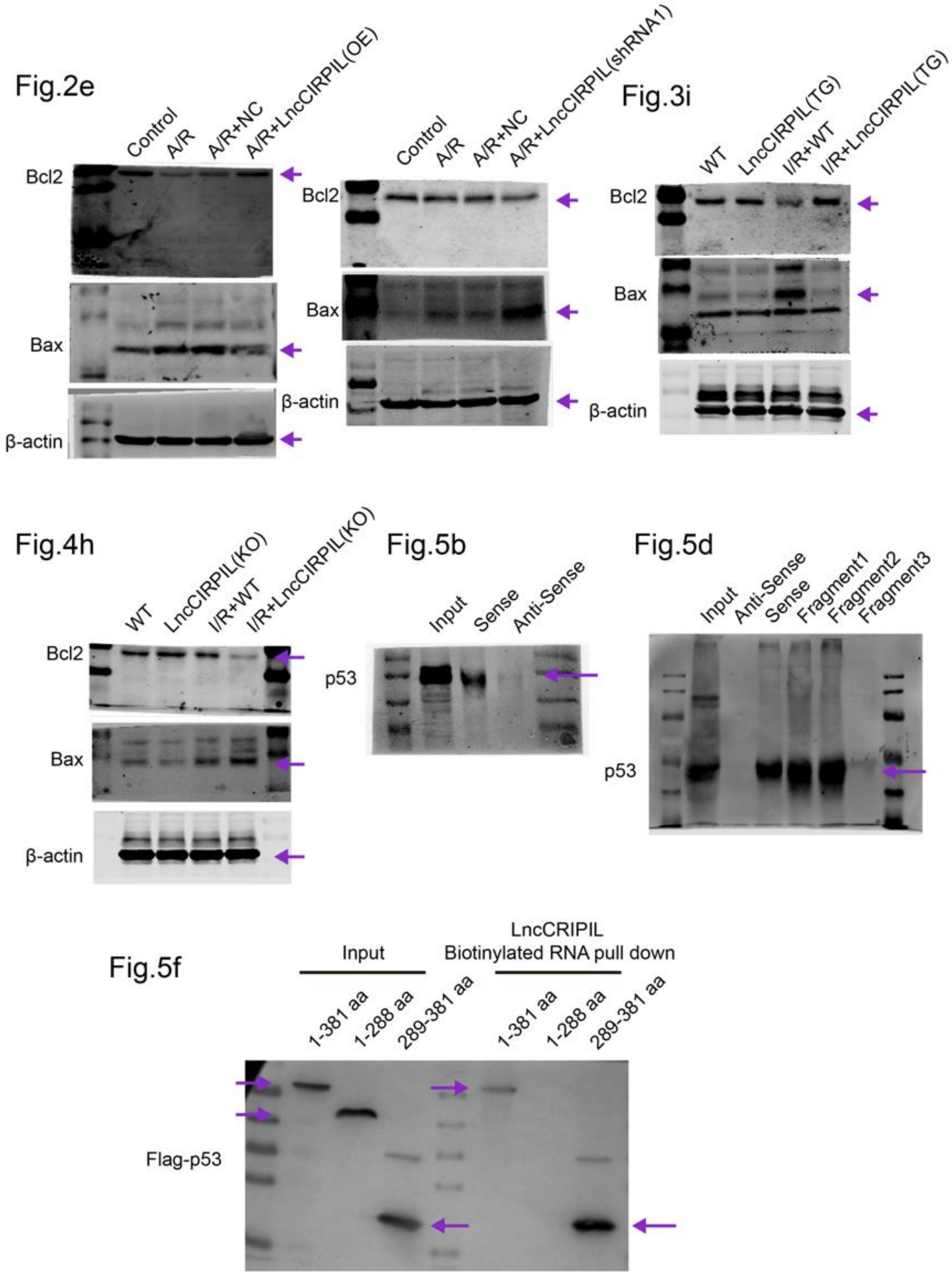

Fig.6b

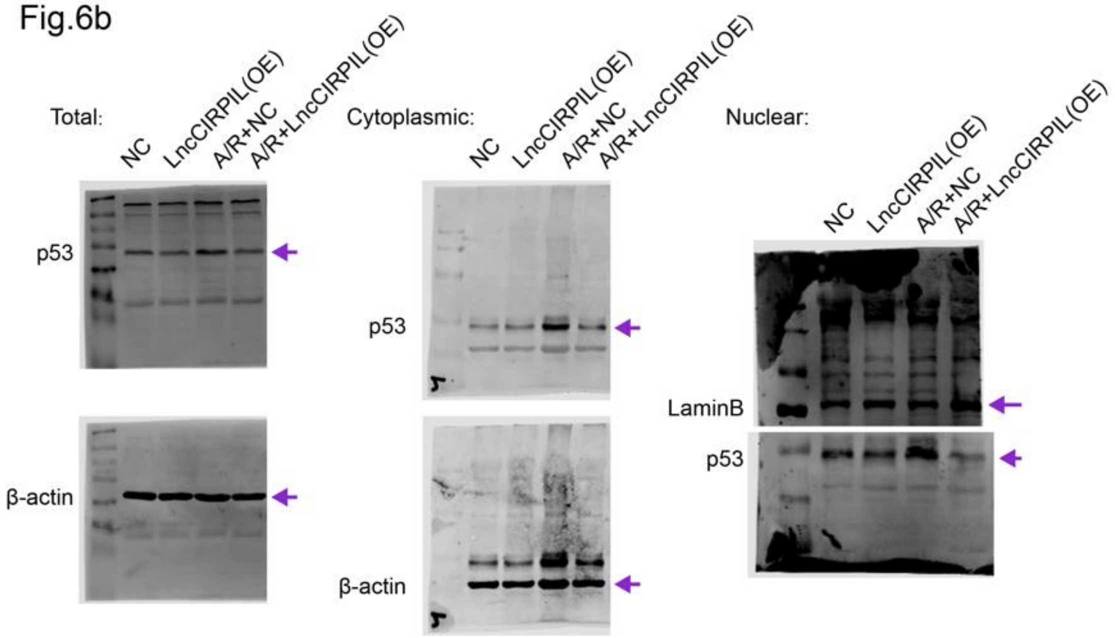

Fig.6d

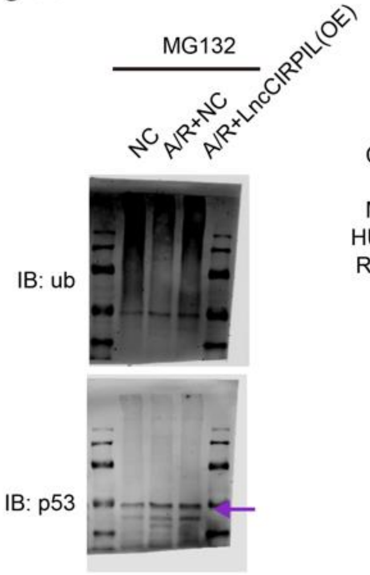

Fig.6f

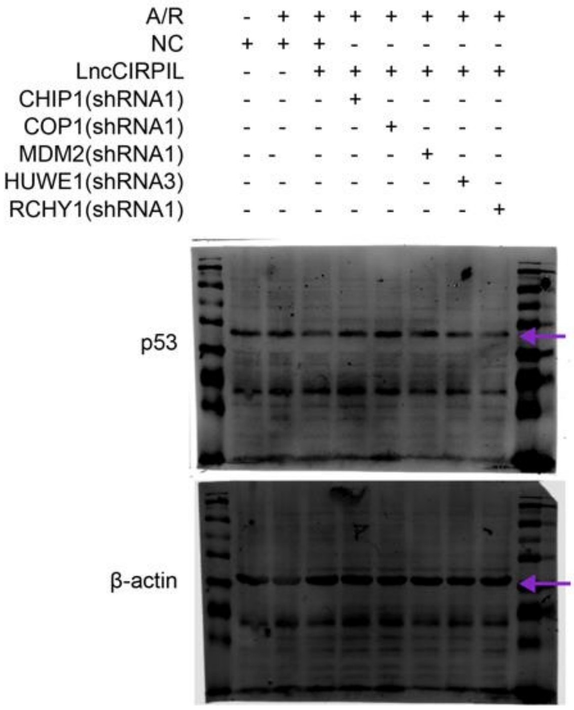

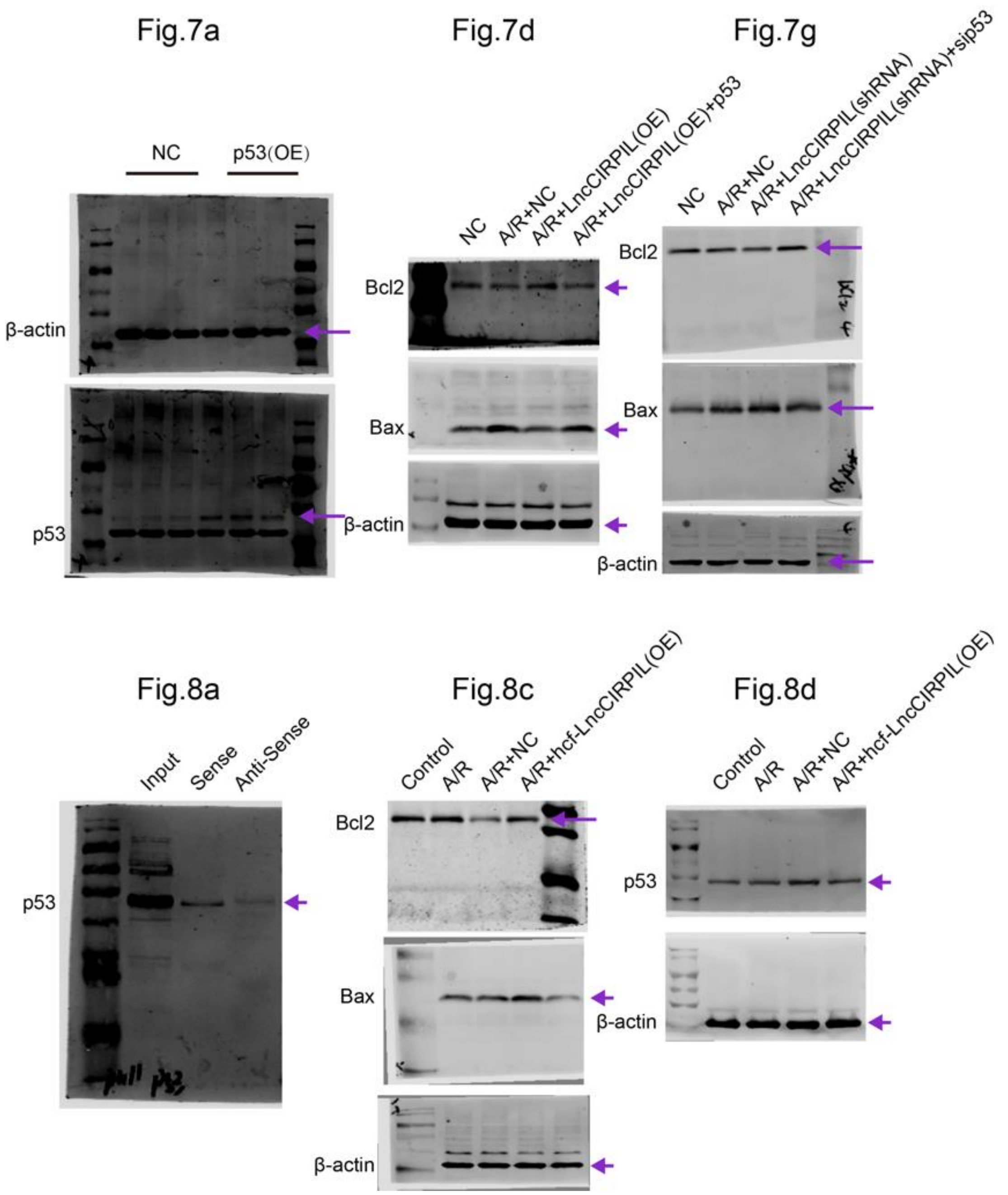
Fig.7a
NC
p53(OE)
β-actin
p53
Fig.7d
NC
A/R+NC
A/R+LncCIRPIL(OE)
A/R+LncCIRPIL(OE)+p53
Bcl2
Bax
β-actin
Fig.7g
NC
A/R+NC
A/R+LncCIRPIL(shRNA)
A/R+LncCIRPIL(shRNA)+sip53
Bcl2
Bax
β-actin
Fig.8a
Input
Sense
Anti-Sense
p53
Fig.8c
Control
A/R
A/R+NC
A/R+hcf-LncCIRPIL(OE)
Bcl2
Bax
β-actin
Fig.8d
Control
A/R
A/R+NC
A/R+hcf-LncCIRPIL(OE)
p53
β-actin

Fig.8g

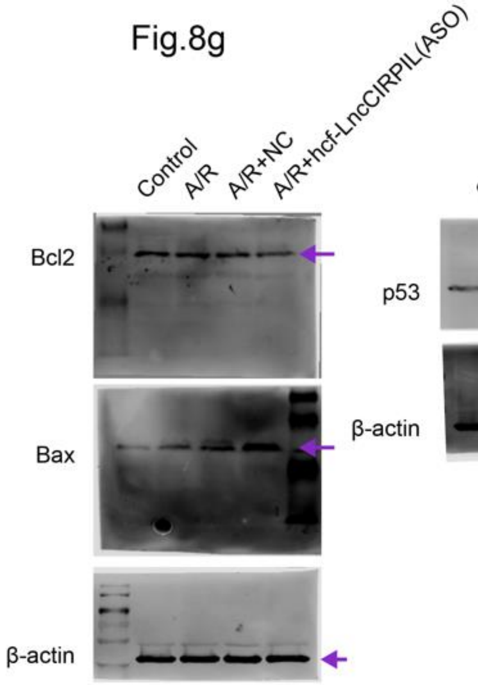

Fig.8h

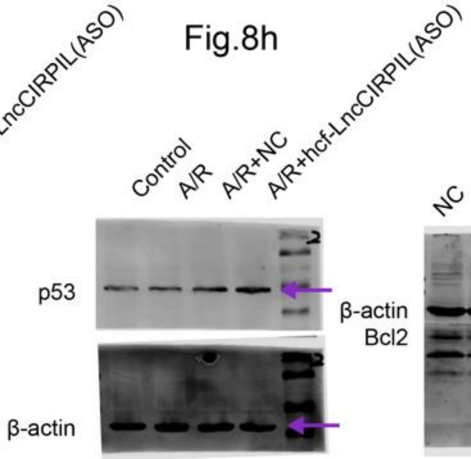

FigS.2e

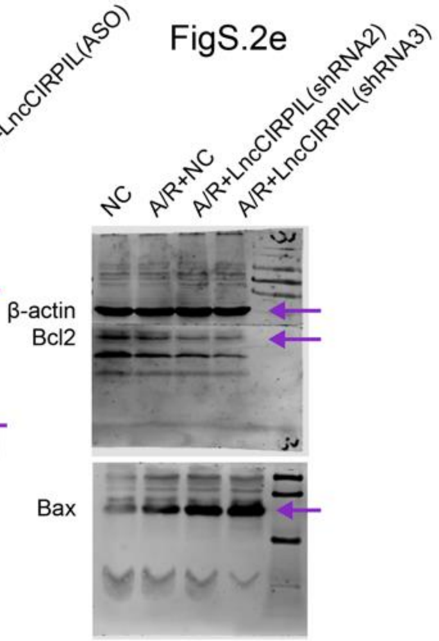

FigS.5b

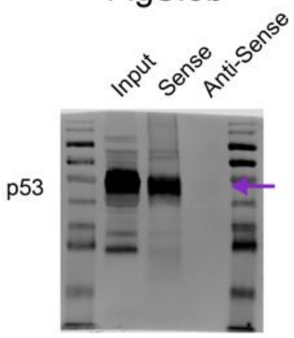

FigS.6b

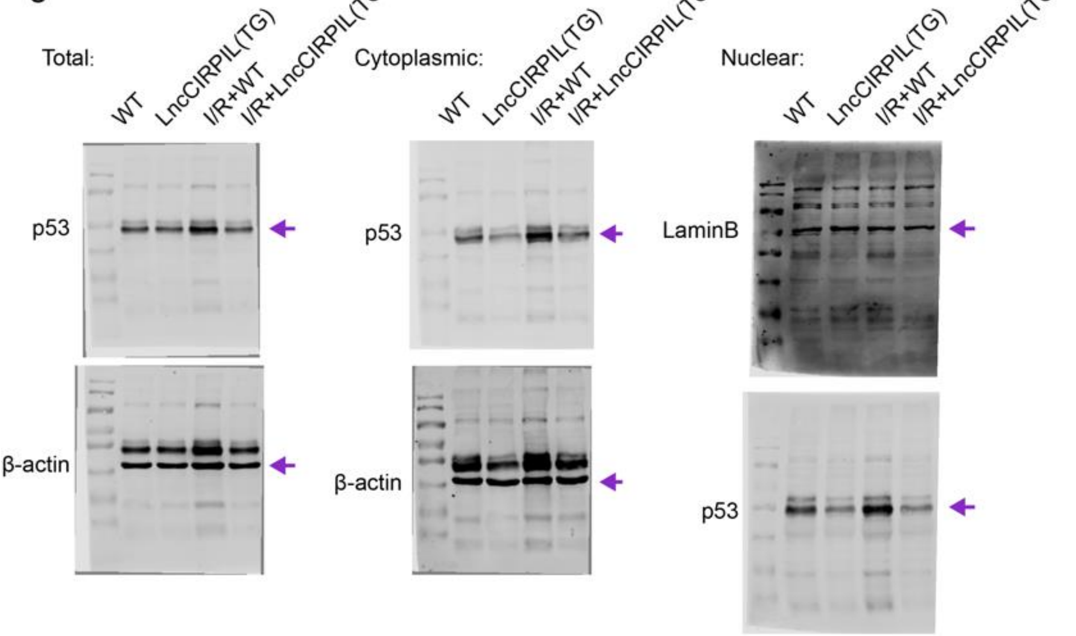

FigS.6d

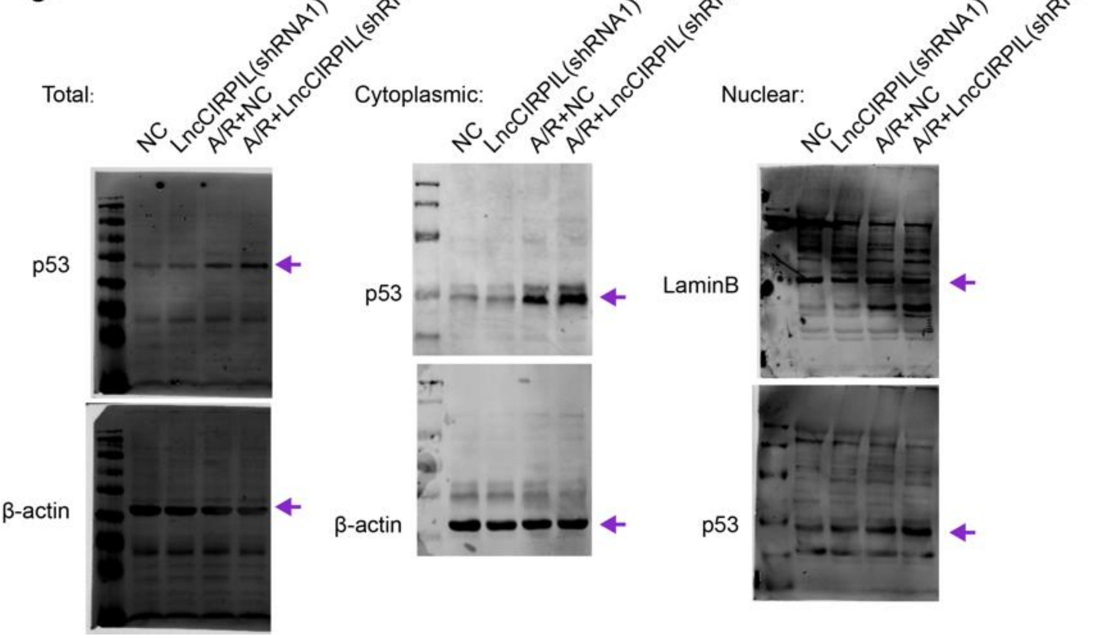

Unprocessed images used for RNA-interacting protein immunoprecipitation assay

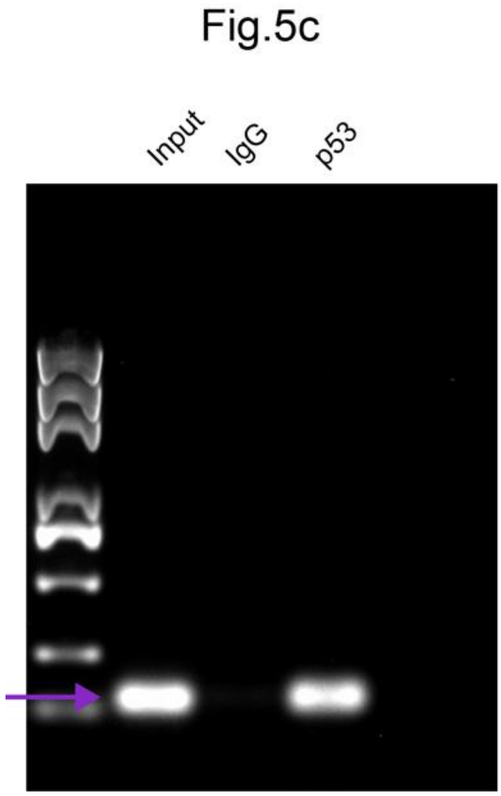

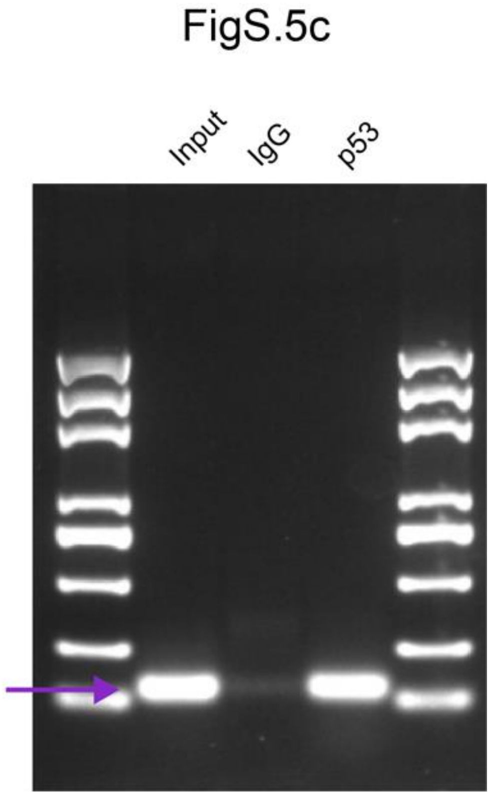

Supplement: Supplementary file 2 — Supplementary Information [file 42003_2022_3651_MOESM2_ESM.pdf]
