# Supplementary material for: Cytoplasmic sequestration of p53 by lncRNA-CIRPILalleviates myocardial ischemia/reperfusion injury
Source: Commun Biol. 2022 Jul 18;5:716. doi: 10.1038/s42003-022-03651-y (PMC9293912; doi:10.1038/s42003-022-03651-y)
Supplement: Supplementary file 5 — Reporting Summary [file 42003_2022_3651_MOESM5_ESM.pdf]

nature portfolio

Corresponding author(s): Zhenwei Pan

Last updated by author(s): Apr 21, 2022

Reporting Summary

Nature Portfolio wishes to improve the reproducibility of the work that we publish. This form provides structure for consistency and transparency in reporting. For further information on Nature Portfolio policies, see our Editorial Policies and the Editorial Policy Checklist.

Statistics

For all statistical analyses, confirm that the following items are present in the figure legend, table legend, main text, or Methods section.

n/a	Confirmed	
☐	☒	The exact sample size (n) for each experimental group/condition, given as a discrete number and unit of measurement
☐	☒	A statement on whether measurements were taken from distinct samples or whether the same sample was measured repeatedly
☒	☐	The statistical test(s) used AND whether they are one- or two-sided<br>*Only common tests should be described solely by name; describe more complex techniques in the Methods section.*
☒	☐	A description of all covariates tested
☐	☒	A description of any assumptions or corrections, such as tests of normality and adjustment for multiple comparisons
☐	☒	A full description of the statistical parameters including central tendency (e.g. means) or other basic estimates (e.g. regression coefficient) AND variation (e.g. standard deviation) or associated estimates of uncertainty (e.g. confidence intervals)
☐	☒	For null hypothesis testing, the test statistic (e.g. F, t, r) with confidence intervals, effect sizes, degrees of freedom and P value noted<br>*Give P values as exact values whenever suitable.*
☒	☐	For Bayesian analysis, information on the choice of priors and Markov chain Monte Carlo settings
☒	☐	For hierarchical and complex designs, identification of the appropriate level for tests and full reporting of outcomes
☒	☐	Estimates of effect sizes (e.g. Cohen's d, Pearson's r), indicating how they were calculated

Our web collection on statistics for biologists contains articles on many of the points above.

Software and code

Policy information about availability of computer code

Data collection	No software was used.
Data analysis	Graphpad Prism 8.0.

For manuscripts utilizing custom algorithms or software that are central to the research but not yet described in published literature, software must be made available to editors and reviewers. We strongly encourage code deposition in a community repository (e.g. GitHub). See the Nature Portfolio guidelines for submitting code & software for further information.

Data

Policy information about availability of data

All manuscripts must include a data availability statement. This statement should provide the following information, where applicable:

- Accession codes, unique identifiers, or web links for publicly available datasets
- A description of any restrictions on data availability
- For clinical datasets or third party data, please ensure that the statement adheres to our policy

The data that support the findings of this study and unique materials are available from the corresponding authors upon reasonable request.

Field-specific reporting

Please select the one below that is the best fit for your research. If you are not sure, read the appropriate sections before making your selection.

☒ Life sciences ☐ Behavioural & social sciences ☐ Ecological, evolutionary & environmental sciences

For a reference copy of the document with all sections, see nature.com/documents/nr-reporting-summary-flat.pdf

Life sciences study design

All studies must disclose on these points even when the disclosure is negative.

Sample size	The sample size for whole animal experiments and molecular biology experiments were set to be >3 for each group.
Data exclusions	The data from the animals died before the completion of the whole experimental procedures were excluded from our data analysis.
Replication	All attempts at replication were successful
Randomization	Animals were randomly divided into control, ischemia-reperfusion injury model model groups, and other groups as specified in the result descriptions.
Blinding	The experimental designers and experimenters/data analysts were double blinded.

Reporting for specific materials, systems and methods

We require information from authors about some types of materials, experimental systems and methods used in many studies. Here, indicate whether each material, system or method listed is relevant to your study. If you are not sure if a list item applies to your research, read the appropriate section before selecting a response.

Materials & experimental systems

n/a	Involved in the study	
☐	☒	Antibodies
☒	☐	Eukaryotic cell lines
☒	☐	Palaeontology and archaeology
☐	☒	Animals and other organisms
☒	☐	Human research participants
☒	☐	Clinical data
☒	☐	Dual use research of concern

Methods

n/a	Involved in the study	
☒	☐	ChIP-seq
☐	☒	Flow cytometry
☒	☐	MRI-based neuroimaging

Antibodies

Antibodies used	p53 (Cell Signaling Technology, 2524), p53 (Proteintech, 10442-1-AP), Bax (Proteintech, 50599-2-lg), Bcl2 (Abclonal, A19693), β-actin (Cell Signaling Technology, 4970), Lamin-B (Abclonal, A1910), LC3I/II (Cell Signaling Technology, 4108), DYKDDDDK Tag (Cell Signaling Technology, 2368), α-actin (Sigma-Aldrich, A2522), IRDye 700CW goat anti-rabbit IgG(H+L) (Licor, 926-68021), IRDye 800CW goat anti-mouse IgG(H+L) (Licor, 926-32210), IRDye 800CW goat anti-rabbit IgG(H+L) (Licor, 926-32211).
Validation	All the primary antibody for the species and application statement on the manufacturer's websites.

Animals and other organisms

Policy information about studies involving animals; ARRIVE guidelines recommended for reporting animal research

Laboratory animals	C57BL/6 adult mice (8 to 10 weeks old) were provided by the Animal Center at the Second Affiliated Hospital of Harbin Medical University. lncCIRPIL cardiac myocyte-specific overexpressing mice, conventional knockout mice were generated by Cyagen Biosciences Inc (Guangzhou, China).
Wild animals	The study does not involve any wild animals.
Field-collected samples	The study does not involve any samples collected from Field.
Ethics oversight	The experimental protocols involving the use of animals in this study were approved by the Animal Care and Use Committee of Harbin Medical University (HMUIRB).

Note that full information on the approval of the study protocol must also be provided in the manuscript.

Flow Cytometry

Plots

Confirm that:

☒ The axis labels state the marker and fluorochrome used (e.g. CD4-FITC).

☒ The axis scales are clearly visible. Include numbers along axes only for bottom left plot of group (a 'group' is an analysis of identical markers).

☒ All plots are contour plots with outliers or pseudocolor plots.

☒ A numerical value for number of cells or percentage (with statistics) is provided.

Methodology

Sample preparation	To quantify cell death by flow cytometry, neonatal mice cardiomyocytes were harvested by digesting with 0.05% trypsin without EDTA after washing in cold PBS. Proteolysis was then neutralized with fetal bovine serum, and the cell suspension were concentrated and resuspended in 100 µL PBS.
Instrument	Backman Coulter, USA, CytoFLEX
Software	CytExpert
Cell population abundance	Only neonatal mice cardiomyocytes were used.
Gating strategy	The live NMCMs were set on the FSC/SSC dot pattern of unstained control cells . And the singlet population were further confirmed by FSC-H/FSC-A dot pattern of unstained control cells. Three cell subpopulations identified from the singlet population of Annexin V-FITC and PI staining A/R cells with unstained control cells as reference.

☒ Tick this box to confirm that a figure exemplifying the gating strategy is provided in the Supplementary Information.
